# Inhibitory coverage of dendritic excitation

**DOI:** 10.1186/1471-2202-12-S1-P291

**Published:** 2011-07-18

**Authors:** Albert Gidon, Idan Segev

**Affiliations:** 1 Department of Neurobiology, The Hebrew University of Jerusalem, Israel; 2 Interdisciplinary Center for Neural Computation, The Hebrew University of Jerusalem, Israel; 3 Edmond and Lily Safra Center for Brain Sciences, The Hebrew University of Jerusalem, Israel

## 

“The Coverage Problem” is a class of optimization problems used in different fields that aims to explore the best strategy in order to maximally “cover” a region under some constrains. For example, how could a limited number of cellular antennas be distributed such that their signals would cover a maximal region (e.g., a whole city) while limiting the radio-frequency radiation to safe levels? Here we ask whether the notion of coverage could be applied to inhibitory synapses, giving them optimal control over the excitatory/excitable activity in the dendritic tree. In support of this idea is the fact that most (85%) [1-3] inhibitory synapses target dendrites rather than operate at the soma/axon region as a global veto mechanism of the neuron’s output . Additionally, it seems to be the rule rather than the exception that single inhibitory axons target specific dendritic sub-regions where each axon makes multiple synapses [4-6], implying that the role of such inhibition is to cover a particular dendritic region. Using analytic solutions of the cable theory as well as detailed compartmental models, we searched for the spatial distribution of inhibition that maximally covers the modeled dendrite under different constraints (e.g., fixed number of contacts per axon and fixed total inhibitory conductance). We explored the conditions in which the inhibitory coverage would be most effective in controlling the neuronal output and compared our results to the actual distributions of inhibitory synapses in different dendrites. We showed that despite the small number of inhibitory synapses (relative to the excitatory synapses) in dendrites of most central neurons, when the synapses are strategically placed, they can effectively dampen the excitatory/excitability activity in dendrites both globally and in a domain-specific manner.

## References

[B1] Beaulieu C Kisvarday Z Somogyi P Cynader M Cowey A Quantitative distribution of GABA-immunopositive and -immunonegative neurons and synapses in the monkey striate cortex (area 17) Cereb. Cortex 1992 2 295 309 10.1093/cercor/2.4.295 1330121

[B2] Beaulieu C Somogyi P Targets and Quantitative Distribution of GABAergic Synapses in the Visual Cortex of the Cat Eur. J. Neurosci 1990 2 296 303 10.1111/j.1460-9568.1990.tb00421.x 12106036

[B3] Liu G Local structural balance and functional interaction of excitatory and inhibitory synapses in hippocampal dendrites Nat. Neurosci 2004 7 373 379 10.1038/nn1206 15004561

[B4] Markram H Toledo-Rodriguez M Wang Y Gupta A Silberberg G Wu C Interneurons of the neocortical inhibitory system Nat. Rev. Neurosci 2004 5 793 807 10.1038/nrn1519 15378039

[B5] Klausberger T Somogyi P Neuronal diversity and temporal dynamics: the unity of hippocampal circuit operations Science 2008 321 53 57 10.1126/science.1149381 18599766 PMC4487503

[B6] Douglas RJ Martin KA Inhibition in cortical circuits Curr. Biol 2009 19 R398 402 10.1016/j.cub.2009.03.003 19467204

